# Lateralization in Reverse Shoulder Arthroplasty

**DOI:** 10.3390/jcm10225380

**Published:** 2021-11-18

**Authors:** Stefan Bauer, Jocelyn Corbaz, George S. Athwal, Gilles Walch, William G. Blakeney

**Affiliations:** 1 Ensemble Hospitalier de la Côte, 1110 Morges, Switzerland; 2 Centre Hospitalier Universitaire Vaudois, 1011 Lausanne, Switzerland; jocelyn.corbaz@gmail.com; 3 Roth/McFarlane Hand & Upper Limb Centre, St. Joseph’s HC, London, ON N6G 4E7, Canada; gsathwal@hotmail.com; 4 Centre Orthopédique Santy, 69008 Lyon, France; gilleswalch15@gmail.com; 5 Royal Perth Hospital, Perth 6000, Australia; blakeney@gmail.com

**Keywords:** Reverse Shoulder Arthroplasty (RSA), lateralization, bipolar lateralization, BIO-RSA, shoulder prosthesis, ROM, notching

## Abstract

Indications for Reverse Shoulder Arthroplasty (RSA) have been extended over the last 25 years, and RSA has become the most frequently implanted shoulder arthroplasty worldwide. The initial Grammont design with medialization of the joint center of rotation (JCOR), placement of the JCOR at the bone–implant interface, distalization and semi-constrained configuration has been associated with drawbacks such as reduced rotation and range of motion (ROM), notching, instability and loss of shoulder contour. This review summarizes new strategies to overcome these drawbacks and analyzes the use of glenoid-sided, humeral-sided or global bipolar lateralization, which are applied differently by surgeons and current implant manufacturers. Advantages and drawbacks are discussed. There is evidence that lateralization addresses the initial drawbacks of the Grammont design, improving stability, rates of notching, ROM and shoulder contour, but the ideal extent of lateralization of the glenoid and humerus remains unclear, as well as the maximal acceptable joint reaction force after reduction. Overstuffing and spine of scapula fractures are potential risks. CT-based 3D planning as well as artificial intelligence will help surgeons with planning and execution of appropriate lateralization in RSA. Long-term follow-up of lateralization with new implant designs and implantation strategies is needed.

## 1. Introduction

The first commercially available and clinically successful reverse shoulder arthroplasty (RSA) was designed by Paul Grammont [1]. Grammont’s RSA design rationale relied on four principles [2]: (I) The joint center of rotation (JCOR) was medialized with a 155° neck-shaft angle (NSA), aiming to increase the lever arm of the deltoid; (II) Placement of the JCOR at the glenoid bone–implant interface for a reduction in shear forces; (III) Deltoid fiber retensioning by distalization of the humerus; (IV) Providing stability by a semi-constrained implant. These principles were successful in allowing stable primary bone–glenoid implant anchorage and in delivering excellent active flexion and abduction. In spite of reasonable clinical results, Grammont’s biomechanical design has a number of downsides such as notching, reduced internal and external rotation, loosening, instability, and loss of contour of the shoulder. Subsequent biomechanical changes have tried to address these drawbacks. Lateralization, which can occur at the glenoid, the humerus or on both sides (global bipolar), has been the focus of the next generation of reverse shoulder implants.

## 2. Traditional Grammont Design

**Figure 1:**
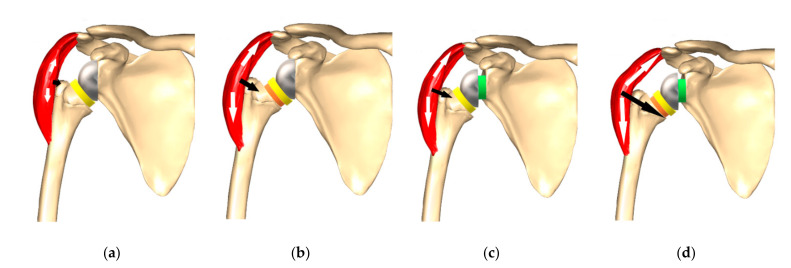
Deltoid wrapping. (**a**) Poor deltoid wrapping due to medialization. (**b**) Improved deltoid wrapping due to humeral lateralization (**c**) Improved deltoid wrapping due to glenoid lateralization. (**d**) Further increase in deltoid wrapping due to combined bipolar lateralization.

The traditional Grammont-style prosthesis is still the most used in many parts of the world and has had excellent long-term clinical outcomes [3,4]. There are, however, some downsides to the design that have been identified. Excessive medialization leads to reduced tensioning of the posterior rotator cuff [5], which may result in instability [6] as well as reduced deltoid wrapping [7] (Figure 1). These factors may explain the slightly higher reported rates of dislocation with traditional Delta III implants, a complication recorded much less in the last 10 years with increased offset biomechanics [5,7,8,9]. Alternatively, the increased dislocation rate may have been due to the learning curve associated with implantation of an RSA, and the expanding indications.

Many studies demonstrate that forward elevation is satisfactorily restored following surgery, but rotation is decreased. Reduced and weak external rotation is caused by a loss of posterior deltoid tension as a result of medialization [5]. Similarly, a loss of anterior deltoid tension leads to decreased and weak internal rotation. Any remaining anterior and posterior cuff may also be less powerful because of the distalization of the humerus, making the vector of pull more oblique.

Humeral medialization and distalization also have the effect of altering the contour of the shoulder due to less offset and more arm length [10]. Finally, an NSA of 155° in combination with medialization of the glenoid leads to higher rates of notching at the scapular margin and pillar [5,11], which can be associated with wear of the polyethylene insert, activation of macrophages and osteoclasts leading to loosening [12,13,14].

## 3. Notching

**Figure 2:**
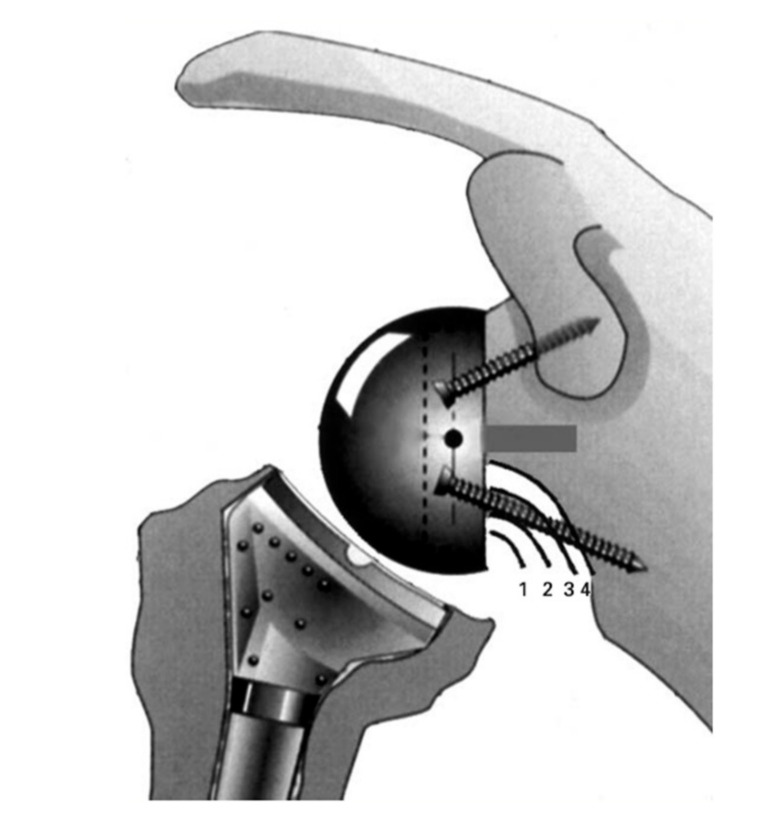
Sirveaux classification of notching [15]. Grade 1, inferior pillar of the scapular neck. Grade 2, the notch is in contact with the lower screw as a result of erosion of the scapular neck to the level of the screw. Grade 3, the notch extends over the lower screw. Grade 4, the notch extends under the baseplate.

Scapular notching was reported to occur in up to 96% of cases, in the orthopedic literature prior to 2010, [15] and was classified by Sirveaux (Figure 2) [16].

New strategies for implant positioning and changes in RSA biomechanics have decreased the occurrence of notching to 10–30% in recent years [14]. Several studies have found that progressive and severe notching is associated with poorer clinical outcomes, reflected by decreased Constant scores [13,16,17,18]. Simovitch reported notching in 14.5% of more than 300 patients with a minimum follow-up of 5 years [19]. The authors showed significantly poorer clinical outcomes, range of motion (ROM), complications and revision rates in patients with notching, in spite of implantation of the baseplate flush with the inferior border of the glenoid and inferior glenosphere overhang. Patient sex and size of the glenosphere (GS) had no impact on rates of notching. The biomechanics and design of the implant used in this study correspond to global bipolar lateralization, as classified by Werthel—25% on the glenoid and 75% on the humerus [8]. RSA with an isolated glenoid-sided offset increase by bone grafting, combined with traditional Delta III stems, has been demonstrated to deliver robust medium to long-term results with 96% graft healing and only 18% with high grade notching [20]. The combination of offset increase at the glenoid with low baseplate position, overhang of the GS as well as a lower NSA with respect to the traditional Grammont prosthesis may lower notching rates in the future. In a meta-analysis of over 2000 RSAs (38 studies) comparing scapular notching between implants with different NSA of 155° and 135° [21], an NSA of 155° was used in 79.3% (n = 1762) of cases, and an NSA of 135° in 20.7% (n = 460) with a lateralized GS. Notching was significantly less frequent (*p* < 0.0001) in the 135° group (2.8%) compared to the 155° group (16.8%).

Stemless reverse shoulder arthroplasty has been reported to have notching in 19% of cases at 2 years [22] and in 72% of cases at 8 years [23]. Such rates of notching are a concern and need further investigation in the future. Associated with high bone-preserving humeral osteotomies, stemless humeral implants may create technical difficulties for the surgeon to adequately retract the humerus and expose the inferior glenoid to implant the baseplate low and flush with the inferior bone. They may also be associated with difficulties with lateralization of the baseplate. For technical as well as for biomechanical reasons, stemless RSA has not been combined with increased glenoid-offset strategies. Stemless RSA is, therefore, currently deprived of the multiple benefits of glenoid-sided lateralization, such as increased stability, increased cuff tensioning, improved deltoid wrapping, greater ROM and reduced notching. The risk of postoperative periprosthetic humerus fractures in the metaphyseal area in up to 10% of cases, due to learning curve issues, requiring stemmed revisions has been presented but not published (DVSE annual conference, Bremen, Germany, 2016) and will need ongoing registry follow-up.

## 4. Lateralization (Global)

Implant lateralization aims to offer better stability by maintaining a state of equilibrium for the length of residual rotator cuff tendons and optimizing the wrapping of the deltoid (wrapping angle). This deviation of the deltoid muscle makes its force vector line cross more medially through the humerus and implant, resulting in increased joint reaction forces (Figure 1) [7,24]. Lateralization should also improve external rotation (ER) by improving the length–tension relationship of infraspinatus and teres minor [7]. Importantly, it should decrease notching by diminished friction in extension (EXT) and ER (friction-type impingement).

**Figure 3:**
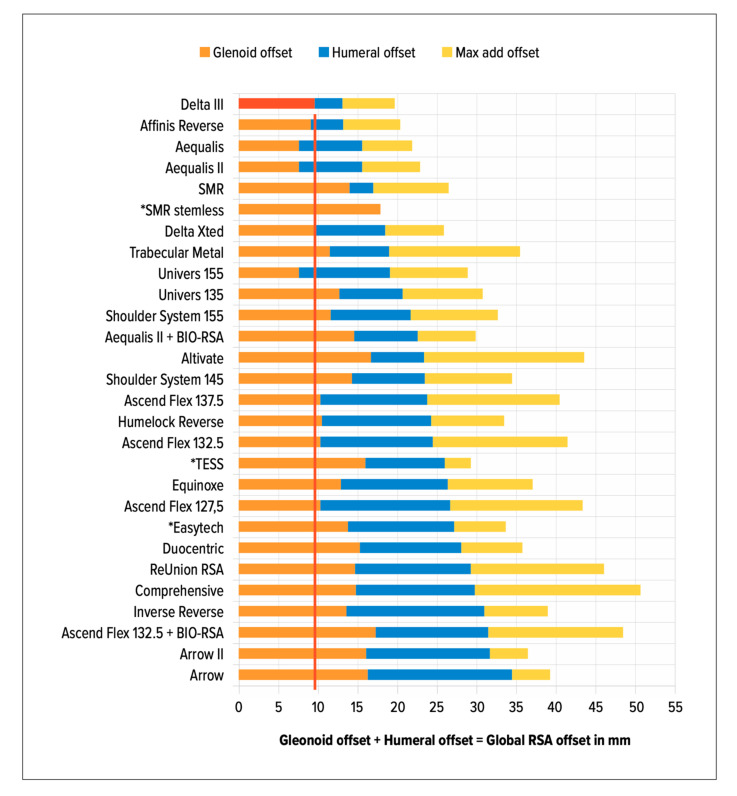
Baseline (red) representing Delta III glenoid-sided lateralization as a reference (* Stemless). Yellow: Maximal additional offset as a result of GS size/other parameters. Figure consistent with Werthel’s data [7].

Whether lateralization of reverse arthroplasty is undertaken on the glenoid or at the humerus has different effects. Werthel et al. studied 28 reverse shoulder implant combinations of 22 different prostheses on the market currently [8]. Grammont’s Delta III prosthesis was used as the baseline reference with 13.1 mm lateralization (global). There was a large variability for global lateralization (global = glenoid + humeral) with a range from 13.1 to 35.8 mm for current prostheses. Global lateralization values were subdivided into the humeral and glenoid contribution. Figure 3 summarizes the breakdown of lateralization for different implants on the market according to Werthel’s article [8].

### 4.1. Glenoid Lateralization

Initial implant designs for lateralization mainly focused on the glenoid. Frankle and his group were the first to lateralize with an increased offset built into the glenosphere [25,26]. This shifts the joint center of rotation lateral to the glenoid bone–implant interface. It also introduces the theoretical risk of failure of fixation due to increased torsional forces at the interface. Biomechanical studies have demonstrated that increased glenoid offset increases baseplate micromotion [27].

**Figure 4:**
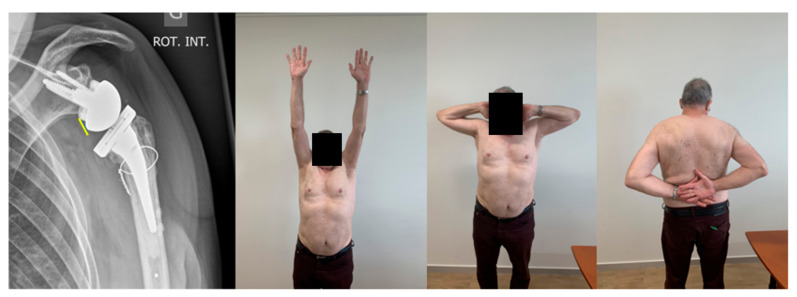
Lateralized RSA after ORIF (Open Reduction Internal Fixation)/osteonecrosis in a young patient after a fracture dislocation and plating. The revision required humeral canal opening with a burr. Outstanding ROM. Radiograph: Combined humeral and glenoid lateralization (Bipolar lateralization; 145° stem, forced varus implantation as a result of sclerosis/insufficient bone stock). Yellow line: Augmented distance from the insert to the lateral scapular margin.

**Figure 5:**
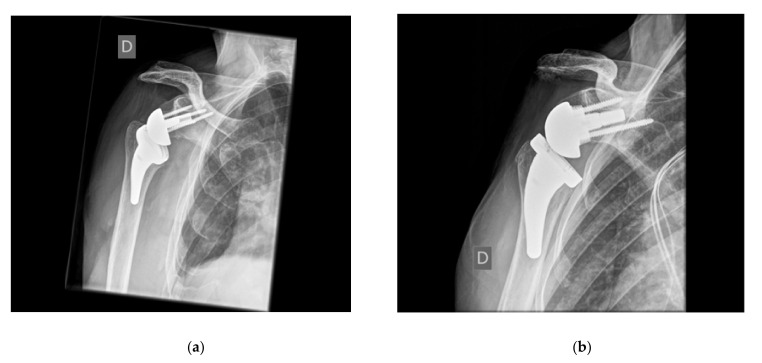
AP radiographs with increased glenoid-sided offset: (**a**) Bony, (**b**) Metal. Minor valgus implantation of stems.

Glenoid-sided lateralization can occur at the baseplate (Figure 4 and Figure 5B), in the glenosphere, and also by lateralizing the baseplate with a bone graft (Figure 5A). This so called bony-increased offset (BIO-RSA) was first popularized by Boileau and has the potential advantage of maintaining the distance from the joint center of rotation to the bone–implant interface after bony integration and union of the graft [28]. A finite element analysis study, however, found that maximum amounts of micromotion for bony ingrowth were reached with only 5 mm of bony lateralization, in comparison to 10–15 mm of glenosphere lateralization [29].

#### 4.1.1. Advantages of Glenoid-Sided Lateralization

A lateralized baseplate reduces impingement with increased ROM (importantly, EXT and ERO) prior to contact with the lateral scapular margin and, therefore, reduces notching (Figure 4) [20,28,30,31]. This was confirmed in a cadaveric study by Berhouet with increasing glenoid offset up to 10 mm [15]. Preserving this glenoid lateralization and increasing the GS diameter from 36 to 42 mm further increased ROM without impingement. Werthel et al., however, demonstrated in their study that changing the GS size from 36 to 42 mm only leads to a global offset increase of +1 mm [8].

Lateralizing the glenoid improves deltoid wrapping and increases joint reaction forces, which may lead to greater stability (Figure 1) [32]. Helmkamp et al. performed a systematic review analyzing post-operative RSA outcomes, comparing medialized versus lateralized glenoid implants. They were able to show significantly better ERO for RSA, which was lateralized on the glenoid side (mean 21° vs. 7°) [33]. The other ROM values were similar. In spite of this, there were no significant differences in clinical scores. The systematic review by Lawrence et al. similarly demonstrated better mean active ERO (46° vs. 24°, *p* < 0.001) for lateralized RSA [34]. They included 349 patients from 13 studies. The incidence of scapular notching was significantly lower in the group with glenoid lateralization compared to traditional glenoid medialization (5.4% vs. 44.9%, *p* < 0.001) [34]. These results are important, given that scapular notching has been found to be associated with worse patient reported outcomes, function, and a higher complication and revision [14,19].

Patients who suffer from a combined loss of active elevation and active external rotation (CLEER) represent a difficult problem compared to a simple loss of active forward elevation (AFE). CLEER can lead to patient dissatisfaction after RSA [16]. Some authors have recommended that a combined tendon transfer (latissimus dorsi/optional teres major) with an RSA should be undertaken to restore ERO as well as elevation [35]. On the other hand, Berglund et al. showed restoration of ERO (from −21° to 28°) in patients with CLEER after glenoid-sided RSA lateralization without a tendon transfer [36].

#### 4.1.2. Possible Negative Effects of Glenoid-Sided Lateralization

Lateralization of the joint center of rotation reduces the moment arm of the deltoid muscle for abduction (ABD) and AFE [24]; therefore, the deltoid muscle force required for ABD rises [37].

Increased shear forces on the interface between the glenoid and the baseplate may increase the risk of loosening [27]. A systematic review reported low rates of clinically important glenoid-sided loosening in traditionally medialized compared to lateralized RSA (1.8% vs. 8.8%, *p* = 0.003) [34]. Zumstein et al. correspondingly published a higher rate of loosening at the glenoid with an RSA using a lateralized JCOR compared to RSA with traditional medialization (5.8% vs. 2.5%, *p* = 0.025) [38]. This study, however, only reported on a single lateralized implant. A more recent systematic review (>6000 RSA from >100 studies) showed only a minor difference in mechanical loosening between lateralized and medialized RSA, without statistical significance (1.15% medialized vs. 1.84% lateralized) [39]. RSA designs with glenoid-sided lateralization may have increased micromotion at the glenoid bone–implant interface, which has been shown in biomechanical studies [27]. We suspect that these differences will likely decrease, as lateralized designs incorporate features that enhance fixation such as larger screws, more screws, and porous coatings.

Lateralized prosthesis designs have been associated with an increased risk of scapular spine and acromial fractures, which may be a result of increased acromial stress. A biomechanical study demonstrated that glenoid lateralization has the greatest effect, increasing stress by 17.2% (for 10 mm of lateralization), compared to 1.7% for humeral lateralization [40]. The study concluded that inferiorization and medialization of the glenosphere were the optimal configuration to minimize acromial stress. They also confirm in their discussion that medialization of the humerus reduces acromial stress. This logical finding can be applied in practice, by medializing the humerus to compensate for glenoid lateralization whilst still making use of its advantages. Haidamous et al., on the other hand, concluded from a multicenter study that distalization had a significantly higher incidence of acromial fractures and they did not detect a negative influence of lateralization [41].

#### 4.1.3. Technical Limitations to Metal-Augmented or Bony Lateralization

The highest metal-augmented glenoid-sided offset on the market according to the evaluation of Werthel was 8.3 mm [8]. This can be enlarged with a BIO-RSA procedure, which uses bone to lateralize the implant. After osseus consolidation, it has the advantage of decreased forces on the bone–implant interface due to a decreased leaver arm [28,42]. However, there are limitations to the extent of lateralization by bone so that secure anchorage is not jeopardized. This normally necessitates a glenoid-sided bone stock of 10–15 mm. It is the authors’ experience that the combination of symmetric grafts up to 10 mm or angled grafts of 7–13 mm (for B, C and E glenoids) with a standard 25 mm baseplate (long peg) and 36 mm eccentric GS using two additional locking screws is safe as long as the RSA angle is corrected with baseplate implantation perpendicular to the supraspinatus fossa line, aiming for a reduction in shear and increase in compressive forces [42].

### 4.2. Humeral Lateralization

Lateralization of RSA on the humerus preserves the reduced torque on the glenoid-sided bone–implant interface of traditional GS medialization, with mechanical gains produced by the global lateralization. Design features of the humeral implant consist of onlay systems, which can be used in onlay but also inlay techniques (more resection), short stems that are curved, and NSA changes from 155° to 135°.

Over the last two decades, the most commonly used NSA for RSA has been 155° [6,13,43,44,45,46,47]. However, a more horizontal humeral implant is mechanically more likely to impinge on the lateral scapular margin. Most recently, implants with NSA of 135° and 145° have been introduced in order to reduce scapular notching by orientating and angling the insert away from the scapular neck. Changing the NSA from 155° to 135° only leads to minimal lateralization of +3.2 mm [8].

#### 4.2.1. Advantages of Humeral Lateralization

Humeral lateralization results in restitution of the position of the humerus compared to Grammont’s design. Hence, the position of the tuberosities is restored, leading to tensioning of the residual rotator cuff [48]. Higher compressive joint reaction forces (JRF) and increased stability are the consequences [49], which are also brought about by increased lateral deltoid tension, the abduction lever arm and wrapping angle of the deltoid (Figure 1) [7]. The moment arms for teres minor, infraspinatus, and the posterior deltoid muscle have been shown to be the most efficient with a lateralized humeral design [50]. A computer simulation study demonstrated that 145° NSA onlay stems with lateralized trays resulted in the greatest lengthening of the supraspinatus and infraspinatus [51].

Humeral lateralization has been shown to decrease the deltoid force required for abduction and has been suggested as a consideration to address the problem of deltoid fatigue [37]. Although, there is no strong clinical evidence for the occurrence of deltoid fatigue.

Biomechanical studies have shown higher ROM with decreasing NSA [52]. A lower NSA raises the ROM before contact with the scapular pillar takes place and lowers the contact area at the inferior margin of the scapula, indicating that less scapular notching would be a likely consequence.

Lower NSAs have also been combined with a modification to platform onlay systems by some manufacturers. The traditional Grammont implant had an inlay metaphysis for an increased metaphyseal bone–implant interface. This, however, leads to loss of metaphyseal bone stock after reaming of the metaphysis. Platform systems in combination with a curved stem maintain metaphyseal bone stock. The angular tilt of the osteotomy may help to prevent damage of the posterosuperior greater tuberosity and rotator cuff. Additionally, the system is adaptable to the necessity to reduce tension by distalizing the osteotomy, whilst still preserving the posterior cuff insertion and using the platform system as an “inlay” prosthesis by positioning the summit of the platform tray at the level of the greater tuberosity. Modularity may also be of advantage in revisions of hemiprostheses and anatomic and reverse shoulder arthroplasty, as easy removal of the platform allows for interchangeability. Platform systems result in variable humeral lateralization according to the position of eccentric onlays [53]. The benefits are restoration of the length of the horizontal rotator cuff muscle tendon units (cuff equilibrium) and lengthening of the moment arm of the deltoid muscle [54]. Reverse shoulder arthroplasty combining an onlay design with a curved stem has demonstrated increased ERO and less notching in comparison to a Grammont-type implant in a clinical trial [55].

#### 4.2.2. Disadvantages of Isolated Humeral Lateralization

A higher incidence of scapular fractures associated with onlay systems has been recorded [55] and confirmed by other observers [56]. Lateralization of the humerus by 5 mm, however, did not significantly raise acromial stress in a biomechanical study [40]. On the other hand, arm over-lengthening with increased tensioning of the deltoid has been attributed as a possible cause of these fractures [57].

Reduced ROM due to impingement with consequent notching may not be as efficiently tackled by humeral- compared to glenoid-sided lateralization. The decrease in notching with lower NSA is primarily caused by the angular tilt of the humeral insert away from the lateral scapular border, rather than its minor lateralization [8].

### 4.3. How Much to Lateralize?

The potential advantages of lateralization come with a risk of excessive global lateralization, with the potential for overstuffing the joint in patients of smaller stature or in patients with contractures of the soft tissues [8,58]. This may lead to difficulties in reducing the joint or with repair of the subscapularis [48]. Additionally, over-lateralization may lead to neuropraxia from nerve stretch injury [8,59,60]. There may be stiffness, reduced range of motion and pain associated with glenoid lateralization. It has also been associated with polyethylene wear, and as discussed, acromial impingement and stress fractures [31].

**Figure 6:**
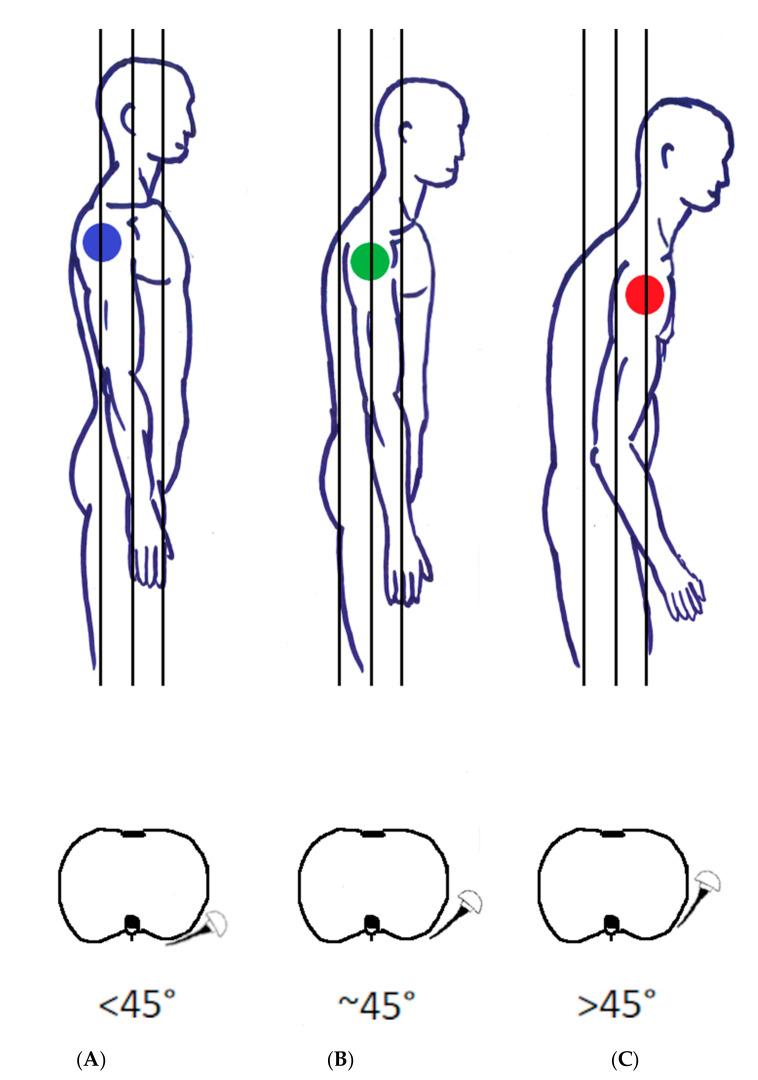
Scapular orientation according to thoracic kyphosis. (**A**) Normal position. (**B**) Mild kyphosis altering scapular orientation. (**C**) Severe kyphosis with pronounced scapular internal rotation.

In patients with thoracic kyphosis, they may have altered scapular orientation with internal rotation, and protraction (anteversion) on the thoracic cage (Figure 6).

**Figure 7:**
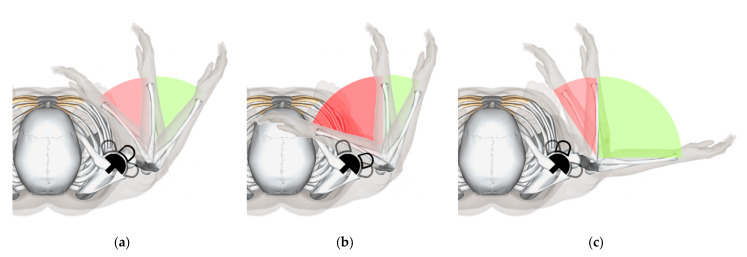
Influence of the humeral implant’s orientation on the ROM. (**a**) Perfect opposition, (**b**) Not enough retrotorsion, (**c**) Too much retrotorsion.

It is important to be aware of scapular orientation prior to RSA, and it has been suggested to adapt retroversion of the humeral implant to match the preoperative scapula orientation (Figure 7) [61]. In our experience, combined retroversion of glenoid and humeral implants may be the best option for some patients with massively increased scapular anteversion and internal rotation >45° secondary to thoracic kyphoses (Type C in Figure 6).

Werthel et al. stated that one of the goals of shoulder arthroplasty should be the anatomical insertion and tensioning of the remaining rotator cuff as well as restoration of an anatomical wrapping angle of the deltoid [8]. To achieve this goal, they recommend greater tuberosity lateralization of 0 mm. The ideal amount of global, glenoid-sided and humeral-sided lateralization and its patient-specific modification, however, remain unknown [11,41,62,63].

## 5. Author’s Suggested Technique

**Figure 8:**
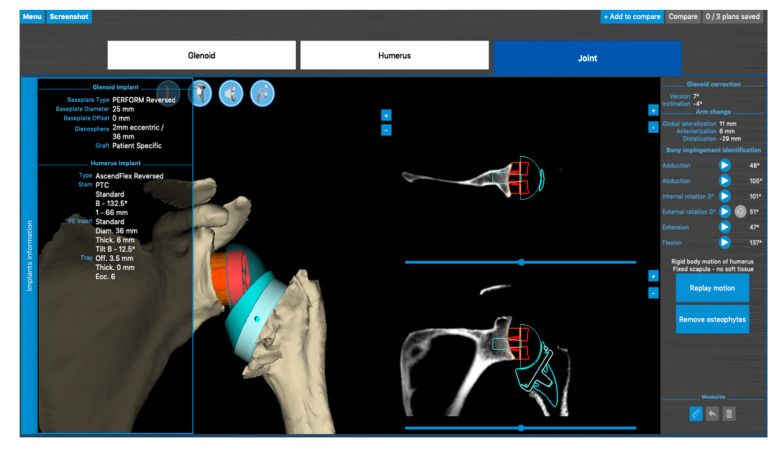
Use of software allowing planning of rigid body motion (Blueprint software, Imascap, Plouzané, France).

The lead author (SB) has developed a technique of patient-specific lateralization, influenced by the literature and discussion with the other authors [2]. All shoulder arthroplasties are planned with 3D planning software (Figure 8), allowing the dynamic simulation and evaluation of impingement-free, rigid body range of motion.

The following parameters displayed in planning software are helpful for the pre-operative plan:

(1) The computed sphere that best fits the 3D model of the glenoid concavity is displayed with the glenoid best fit sphere radius (GBFSR), a marker of the glenohumeral size (observed range: 25–45 mm). Patients with GBFS of less than 30 mm are at higher risk of overstuffing.
(2) Lateralization (variation 0 to +10 mm): The surgeon should be cautious with lateralization beyond +5–10 mm depending on the amount of loss of medial bone stock. Excessive superior migration (vertically decentered) but, more importantly, posterior (e.g., B2 glenoid) and anterior subluxation are signs that there will be increased tension when attempting to reduce the RSA.
(3) Distalization (variation: 20 to +40 mm) depends on the amount of cranial humeral head migration. The surgeon should be cautious with distalization beyond 35–40 mm.

The author’s (SB) case series of more than 150 RSA (which has not yet been published) shows that computed preoperative rigid body motion aiming to prevent notching with ERO and EXT of at least 45° can, in the majority of cases, only be accomplished by increased glenoid-sided lateralization.

### 5.1. Glenoid Side

In the majority of cases, a MIO (metal-increased offset)- or BIO-RSA with +3–10 mm of lateralization is used. It is the author’s preference to lateralize with bone (BIO-RSA) whenever possible. A 25 mm baseplate is preferred for sufficient inferior overhang, implanted flush with the inferior margin of the glenoid respecting the RSA angle, with implantation of the baseplate perpendicular to the supraspinatus fossa line [42]. A 36 mm eccentric (+2 mm) GS is used for the majority of patients, with some exceptions for male patients of larger stature. This strategy aims to maintain the subacromial space for ERO in elevation and to simultaneously reduce friction-type impingement in ERO and EXT.

### 5.2. Gap Space Assessment

**Figure 9:**
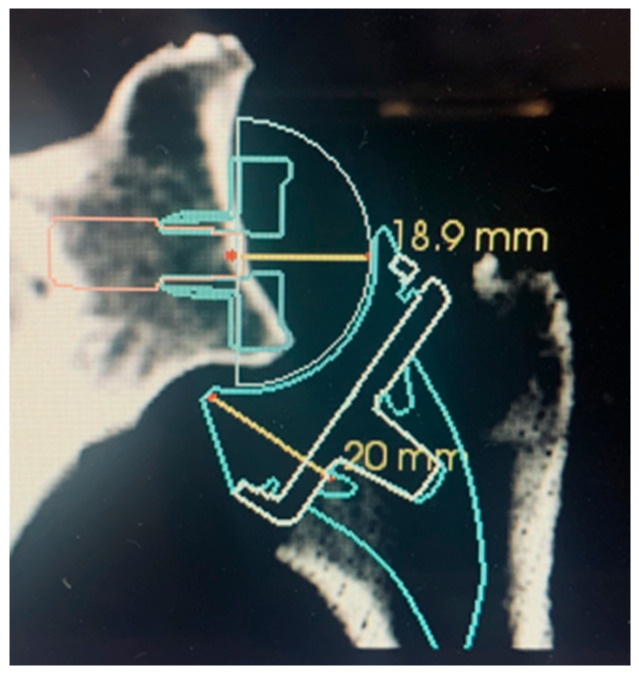
Gap space requirement for reduction of a standard 36 mm GS with a standard insert (+6 mm) and tray (+0 mm). Baseplate/GS distance of approximately 18.9 mm and insert/tray jump distance of 20 mm.

Provided the osteotomy of the humerus is considered definitive, the extent of possible glenoid offset can be judged with a plastic spacer. The distance between the glenoid surface after reaming and the osteotomy of the humerus requires approximately 39 mm for reduction in the components. This distance can be divided into 18.9 mm (25/36 baseplate/glenosphere) +20 mm (humeral insert height), as illustrated in Figure 9. A gap space assessment can be undertaken after glenoid reaming by inserting a 40 mm plastic spacer with a handle to assess the gap tension. Adequate lateralization with metal (the author’s standard baseplate already has +2 mm inbuilt offset with optional augmentation of +3 and +6 mm) or bone (±2 mm baseplate +10 mm bone) can be evaluated. These absolute values depend on different systems on the market but can be calculated for each RSA design.

### 5.3. Humeral Side

The author’s preference is to adapt the humerus to balance increased glenoid-sided lateralization. After implantation of increased glenoid offset, it is often necessary to recut or ream the humerus, leading to a metaphyseal inlay seating of the platform. The lateral summit of the platform tray is aligned at the level or a few millimeters below the greater tuberosity. This technique of humeral compensation for increased tension is possible with onlay systems implanted in the inlay technique, but cannot be applied to all stems and metaphyseal implants. Because of the low osteotomy, a +3.5 mm eccentric tray, which is dialed to position 6, is, in most cases, necessary for coverage. This medializes the humerus by 3 mm (2 + 1 mm). After implantation of the GS, the necessary amount of humeral resection can be evaluated a second time with a 20 mm plastic spacer. Under muscle relaxation, its insertion between the GS and the surface of the osteotomy should be possible. These measures reduce tension by medialization and adaptive shortening of the humerus, allowing for an increase in the glenoid neck length with its known benefits. This adaption reduces the risk of acromion fractures [41]. Humeral trial inserts with integrated pressure sensors have been developed by one company; however, the ideal tension and joint reaction force after reduction of an RSA in a patient under muscle relaxation is currently unknown.

### 5.4. Risks

After glenoid-sided lateralization and humeral compensatory resection, the reduction of the RSA can still be difficult and even impossible, if no initial assessment of the gap space between the reamed glenoid surface and humeral osteotomy was carried out. A plastic prosthesis reducer is useful to overcome the “jump distance” of the humeral insert concavity (Figure 9, 20 mm) as well as removal of the arm out of the armholder, with the arm being free of any constraints. The situation of impossible reduction, where the required compensatory humeral osteotomy would be too low at the humeral calcar, can occur. This extremely rare scenario should be addressed by intraoperative removal of the GS and baseplate with a reduction in the glenoid-sided offset. The humeral osteotomy and inlay strategy should also never compromise the insertion of the posterior rotator cuff. Additional inlay reamers have been designed for some platform onlay systems to prevent posterolateral over-resection and damage of the posterior cuff.

## 6. Conclusions and Outlook

Reverse shoulder arthroplasty has been providing excellent long-term clinical outcomes and survivorship, leading to increasing worldwide use. Newer designs have addressed some of the problems of notching, decreased internal and external range of motion and instability by lateralization. Lateralization at the glenoid has its own drawbacks of increased shear forces on the baseplate, increased acromial stress and decreased moment arm of the deltoid. Lateralization at the humerus with changes in neck shaft angle may also lead to increased acromial fractures and may not avoid notching as effectively as glenoid lateralization. In the near future, artificial intelligence may help to determine the optimal combination of lateralization, glenosphere size, eccentricity and distalization to achieve the best specific and global ROM. Further prospective clinical studies are also needed to confirm the benefits on patient outcomes.

## Data Availability

Not applicable.
